# Kv3.4 regulates cell migration and invasion through TGF-β-induced epithelial–mesenchymal transition in A549 cells

**DOI:** 10.1038/s41598-024-52739-4

**Published:** 2024-01-28

**Authors:** Hun Ju Sim, Mi Ri Kim, Min Seok Song, So Yeong Lee

**Affiliations:** 1 Laboratory of Veterinary Pharmacology, College of Veterinary Medicine and Research Institute for Veterinary Science, Seoul National University, 1 Gwanak-ro, Gwanak-gu, Seoul, 08826 Korea; 2 Department of Physiology, College of Medicine, Gyeongsang National University, Jinju, 52727 Korea

**Keywords:** Potassium channels, Receptor pharmacology

## Abstract

Epithelial–mesenchymal transition (EMT) is the process by which epithelial cells acquire mesenchymal characteristics. This process induces cell migration and invasion, which are closely related to cancer metastasis and malignancy. EMT consists of various intermediate states that express both epithelial and mesenchymal traits, called partial EMT. Recently, several studies have focused on the roles of voltage-gated potassium (Kv) channels associated with EMT in cancer cell migration and invasion. In this study, we demonstrate the relationship between Kv3.4 and EMT and confirm the effects of cell migration and invasion. With TGF-β treatment, EMT was induced and Kv3.4 was also increased in A549 cells, human lung carcinoma cells. The knockdown of Kv3.4 blocked the EMT progression reducing cell migration and invasion. However, the Kv3.4 overexpressed cells acquired mesenchymal characteristics and increased cell migration and invasion. The overexpression of Kv3.4 also has a synergistic effect with TGF-β in promoting cell migration. Therefore, we conclude that Kv3.4 regulates cancer migration and invasion through TGF-β-induced EMT and these results provide insights into the understanding of cancer metastasis.

## Introduction

Cancer metastasis is closely involved in cancer-related deaths, especially since more than 90% of cancer mortality is related to metastasis^1–3^. One of the major mechanisms of cancer metastasis is epithelial–mesenchymal transition (EMT)^4^. EMT is important not only for embryonic development and tissue repair but also for cancer metastasis^5^. During EMT, epithelial cells lose their characteristics, such as apical–basal polarity and cell–cell junctions, and gain mesenchymal phenotypes and motility^6^. In general, EMT is accompanied by morphologic and molecular changes. Morphology is altered from cuboidal or columnar shapes to elongated and spindle-like phenotypes. In addition, the expressions of epithelial markers, such as E-cadherin, are decreased; in contrast, mesenchymal markers, including N-cadherin and vimentin, are increased^7^. This process is closely involved in cancer migration and invasion, and is regarded as the first step in the metastatic cascades^1,4–7^. Recently, EMT has not been considered a binary phase. EMT consists of various degrees of partial and intermediate phases coexpressing epithelial and mesenchymal traits, which reflects cellular plasticity and diversity. This process is called partial EMT^6,8–10^.

Voltage-gated potassium (Kv) channels are considered novel targets in cancer and are particularly related to cancer metastasis. Indeed, upregulated Kv channels are correlated with cancer malignancy, such as Kv1.3^11^, Kv2.1^12^, Kv10.1^13^, and Kv11.1^14^. Recent studies have found that Kv3 channels, including Kv3.4, are related to vimentin in human and canine tumor cells^15^ and promote cancer cell migration and invasion^16^. In addition, higher expression levels of Kv3.4 are associated with more aggressiveness in laryngeal^17^, oral^18^, and head and neck squamous cell carcinoma^19^. Thus, Kv3.4 is considered to be related to cancer malignancy.

The relationship between Kv channels and EMT has been shown, such as Kv7.1^20^, Kv10.1^21^, Kv11.1^14,22,23^. However, the precise involvement between Kv3.4 and EMT is still unclear. Therefore, using transforming growth factor-β1 (TGF-β), a representative EMT inducer^24^, we confirmed the expression levels of Kv3.4 in TGF-β-induced EMT. We investigated the effects by the regulation of Kv3.4 on cell morphology, migration and invasion, and related EMT markers changes in A549 cells. Thus, the present study focuses on the role of Kv3.4 in TGF-β-induced EMT using A549 cells and its association with Kv3.4 and cancer malignancy.

## Results

### TGF-β induces EMT and increases the expression levels of Kv3.4 in A549 cells

**Figure 1:**
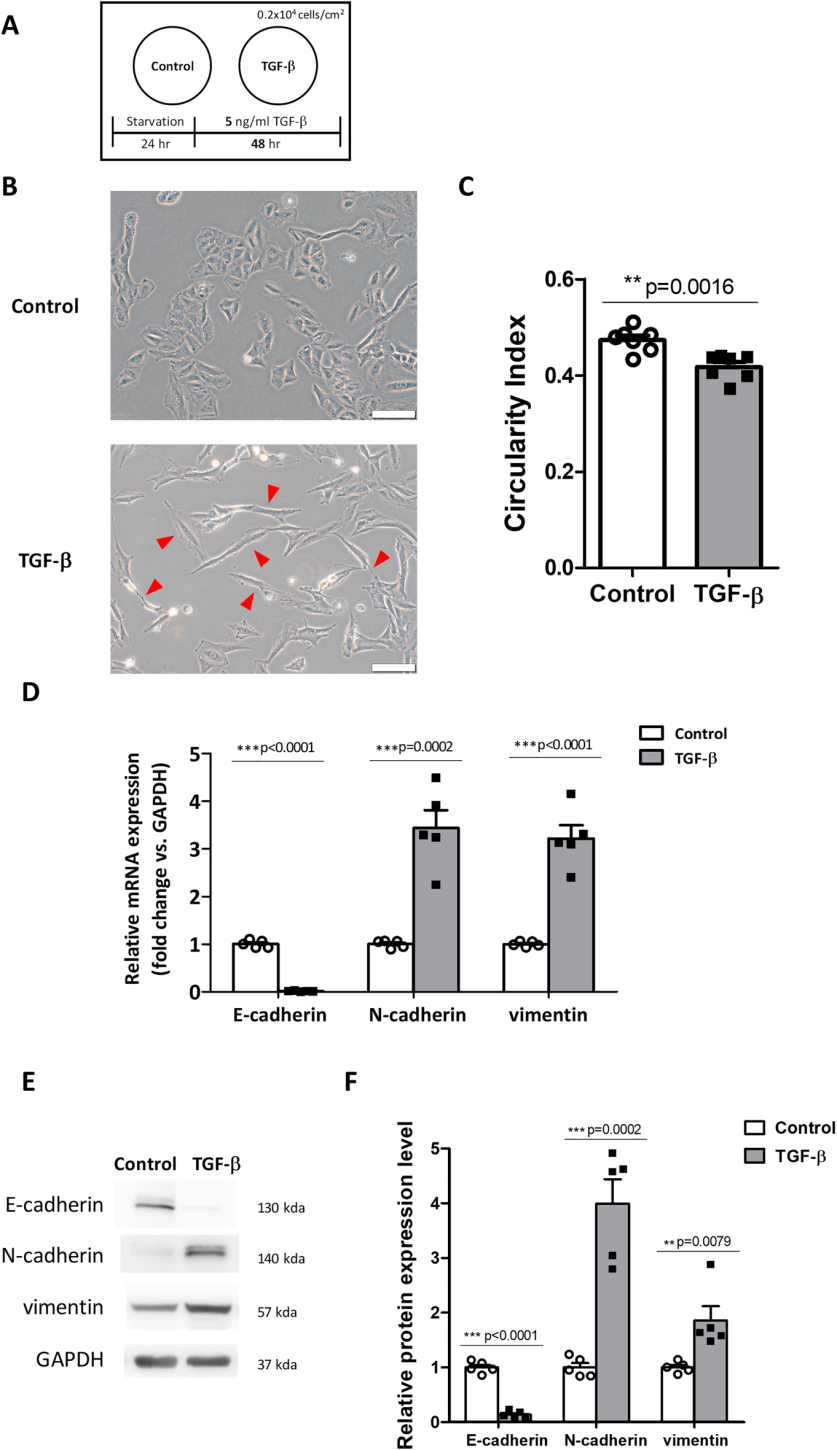
TGF-β induced EMT in A549 cells. (**A**) Schematic diagram demonstrates the experimental design of TGF-β treatment in A549 cells. (**B,C**) Representative phase-contrast images and the circularity index graph show morphological changes with 5 ng/ml TGF-β treatment for 48 h in A549 cells (×200). Scale bar 100 μm. Arrow pointing to elongated form in the TGF-β treatment group. (**D–F**) TGF-β treatment altered the molecular levels of EMT markers (E-cadherin, N-cadherin, and vimentin) in mRNA (**D**) and protein (**E**,**F**) levels. All experiments were repeated (n = 5, except for in (**C**), n = 7). The data represent the mean ± standard error. **p < 0.01; ***p < 0.001 (unpaired *t* test (**D**,**F**) and Mann–Whitney U test (**F** for vimentin)).

**Figure 2:**
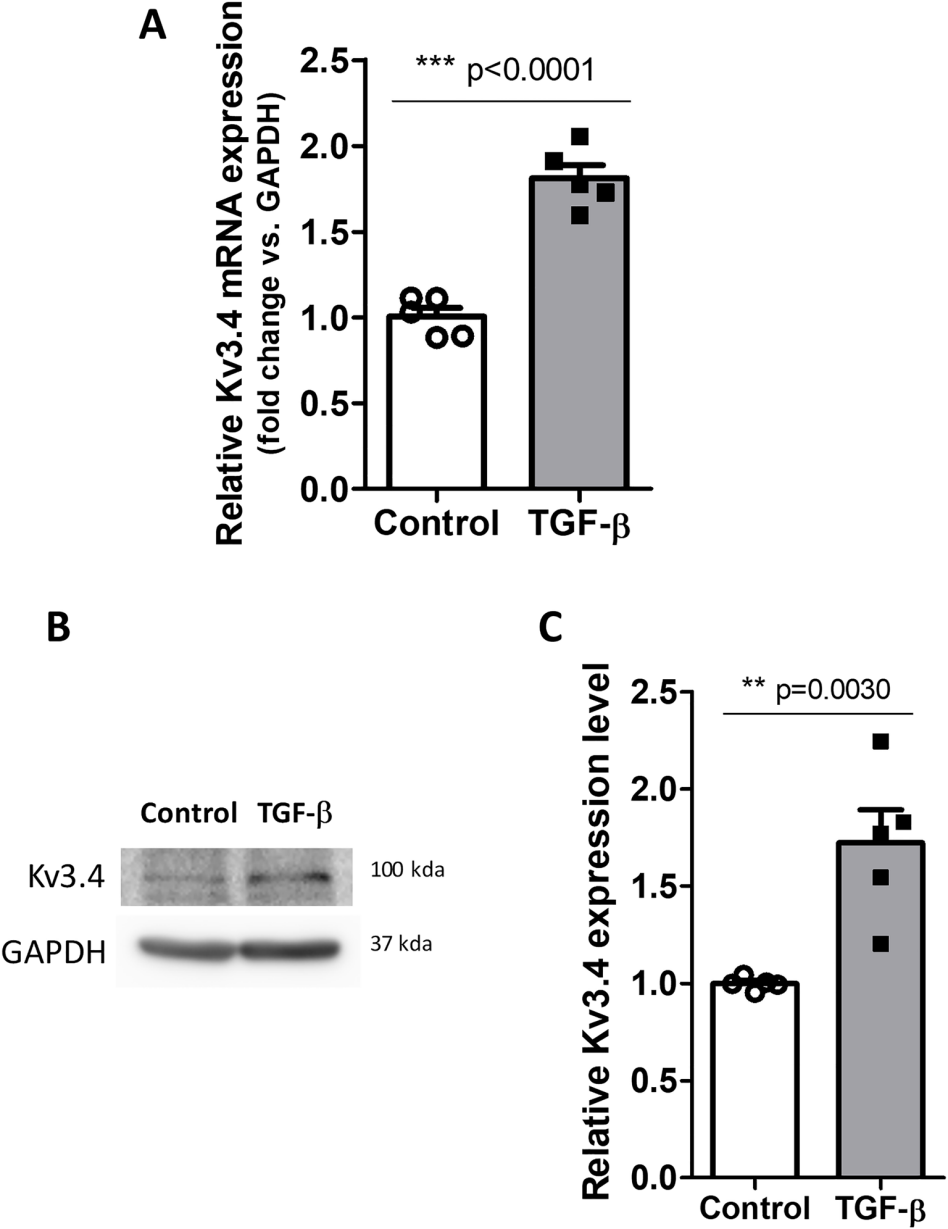
TGF-β treatment increased the expression levels of Kv3.4. (**A**) Representative graph shows the expression level of Kv3.4 in mRNA level after treatment with 5 ng/ml TGF-β for 48 h in A549 cells. (**B**,**C**) The protein level of Kv3.4 in A549 cells was analyzed by western blot (**B**) and the quantitative graph (**C**) after the TGF-β treatment. All experiments were repeated (n = 5), and the data represent the mean ± standard error. **p < 0.01; ***p < 0.001 (unpaired *t* test).

To confirm the relationship between Kv3.4 and EMT, we treated 5 ng/ml of TGF-β for 48 h in A549 cells to induce EMT (Fig. 1A)^24^. TGF-β treatment does not affect cell viability (Supplementary Fig. 1A). Compared to the cuboidal and bundled shapes of control, TGF-β treated cells had elongated spindle shapes and exhibited a lower circularity index (Fig. 1B,C). TGF-β treatment also changed the molecular levels of EMT markers (Fig. 1D–F). E-cadherin, a representative epithelial marker, was significantly reduced. In contrast, N-cadherin and vimentin, mesenchymal markers, apparently increased in both mRNA and protein levels. In addition, we assessed whether TGF-β treatment affected the expression levels of Kv3.4 (Fig. 2A–C). Our data showed that the mRNA and protein levels of Kv3.4 significantly increased in A549 cells.

### The knockdown of Kv3.4 blocks EMT progression with reducing cell migration and invasion

**Figure 3:**
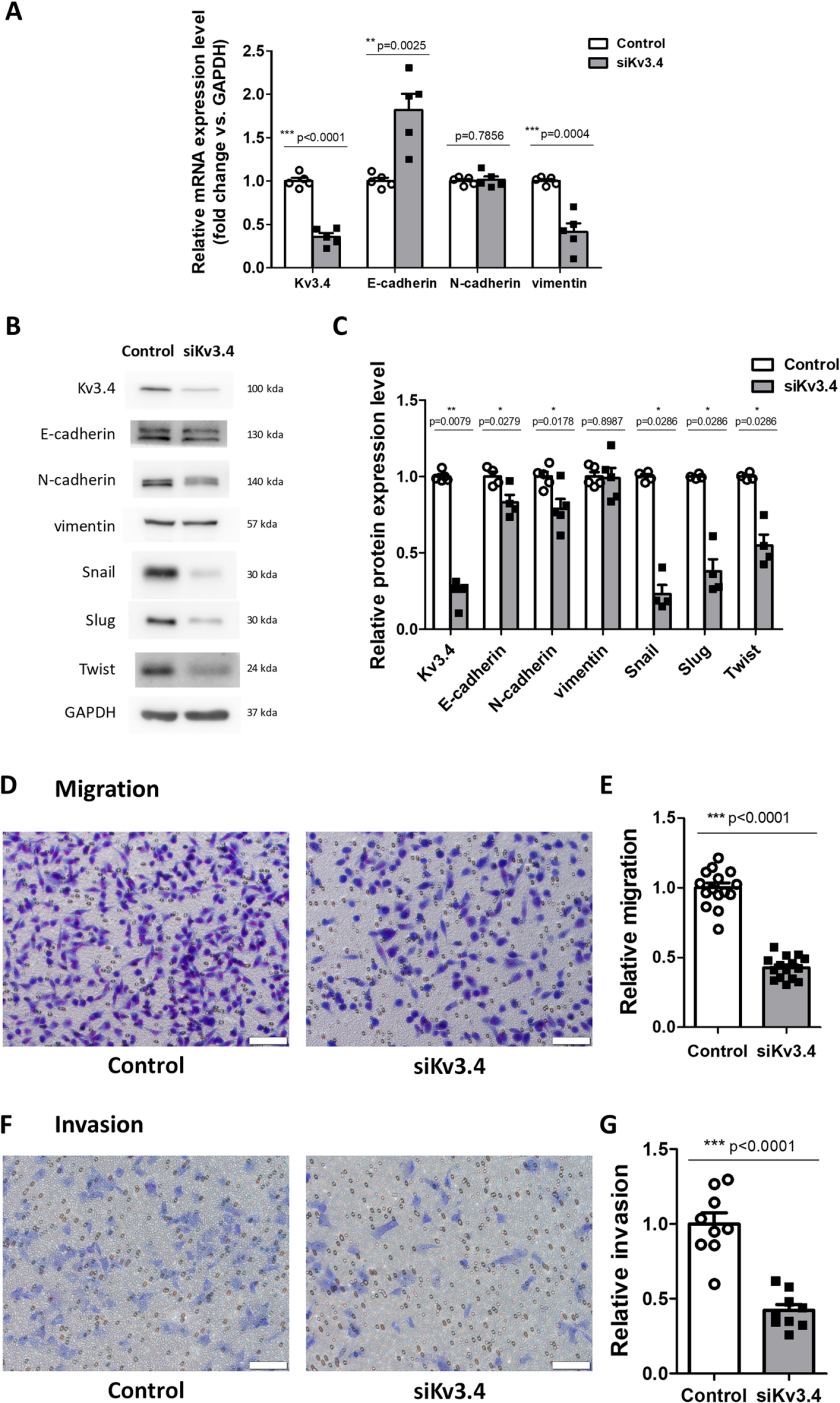
The knockdown of Kv3.4 blocked EMT progression with reducing cell migration and invasion. (**A**) After 50 nM of siKv3.4 treatment for 72 h, the expression changes of Kv3.4, E-cadherin, N-cadherin, and vimentin in mRNA levels. (**B**,**C**) The expression levels of Kv3.4, E-cadherin, N-cadherin, vimentin, Snail, Slug, and Twist were analyzed by western blot after siKv3.4 treatment. (**D–G**) Representative images and graphs show the effects of siKv3.4 treatment on cell migration (**D**,**E**) for 24 h and invasion (**F**,**G**) for 48 h compared with Control (×200). Scale bar 100 μm. The experiments were repeated ((**E**,**G**): n = 3, E-cadherin, Snail, Slug, Twist in (**C**): n = 4, (**A**,**C**): n = 5) and the data represent the mean ± standard error. *p < 0.05; **p < 0.01; ***p < 0.001 (unpaired *t* test (**A**,**C**,**E**,**G**), Mann–Whitney U test (**C** for Kv3.4, Snail, Slug, Twist)).

Using small interfering RNA of Kv3.4 (siKv3.4), we investigated the knockdown effect of Kv3.4. After 50 nM of siKv3.4 for 72 h in A549 cells, we examined the changes in EMT markers on mRNA and protein levels (Fig. 3A–C). The transfection of siKv3.4 did not affect cell viability (Supplementary Fig. 1B). In mRNA levels, E-cadherin, an epithelial marker, was significantly increased, but vimentin, a mesenchymal marker, was remarkably decreased. N-cadherin, a mesenchymal marker, was not altered in the mRNA levels (Fig. 3A). On the other hand, according to the western blot data, the expression levels of E-cadherin and N-cadherin were slightly decreased, but vimentin expression did not change. In addition, the expression levels of the transcriptional factors such as Snail, Slug, and Twist were significantly reduced (Fig. 3B,C). From these results, we conclude that the reduction of Kv3.4 decreases the mesenchymal markers coexisting with E-cadherin. To further investigate the functions of siKv3.4, we performed the transwell migration and invasion assay after siKv3.4 treatment. Our data showed that siKv3.4 treatment significantly inhibited cell migration and invasion (Fig. 3D–G).

### The overexpression of Kv3.4 acquires mesenchymal characteristics and enhances cell migration and invasion

**Figure 4:**
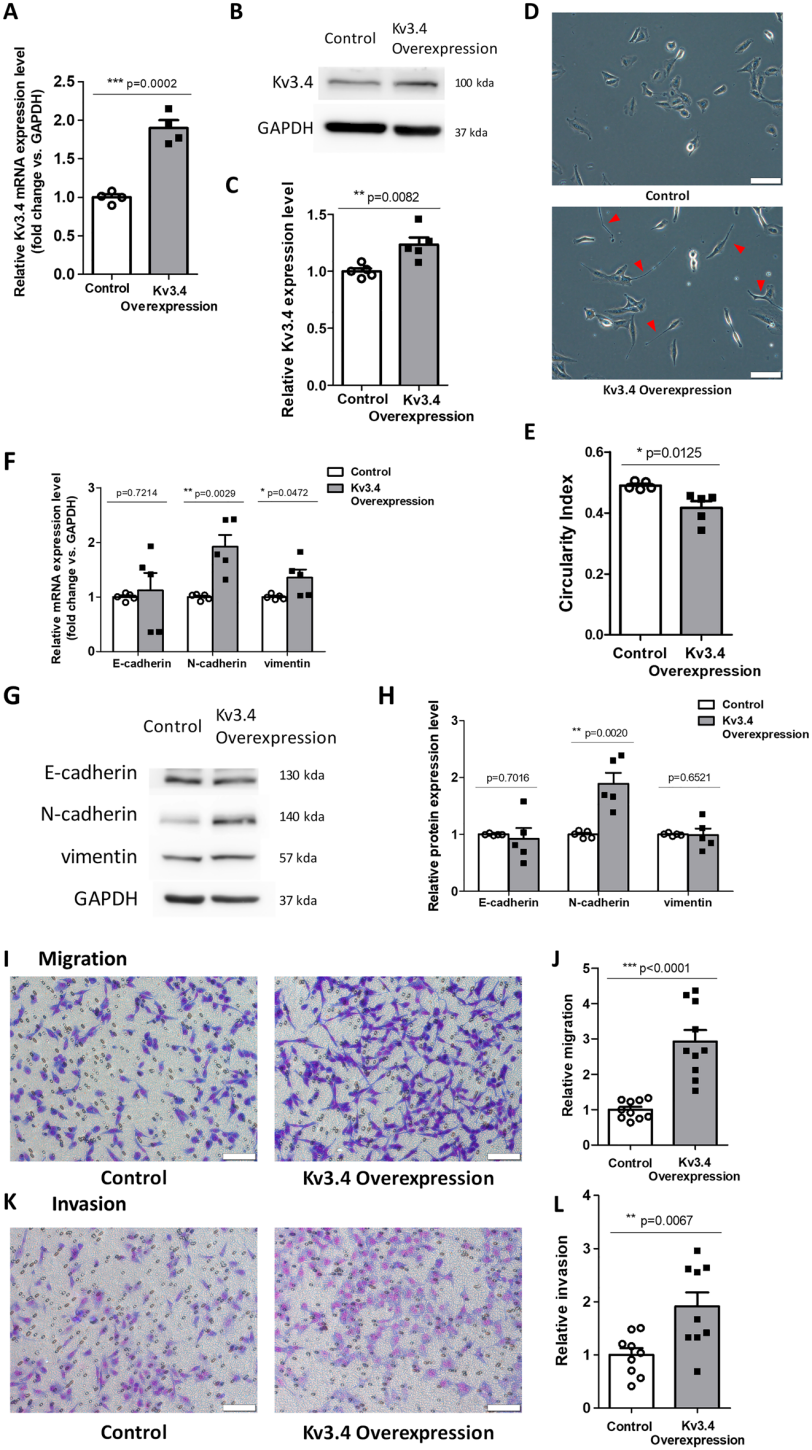
The overexpression of Kv3.4 acquired mesenchymal characteristics and enhanced cell migration and invasion. (**A**–**C**) The overexpression of Kv3.4 confirmed in mRNA (**A**), western blotting (**B**) and quantitative graph of protein levels (**C**) in A549 cells. (**D,E**) Cell morphology of Control and Kv3.4 overexpressed cells in representative images (**D**) and the circularity index graph (**E**) (×200). Scale bar 100 μm. (**F–H**) The effects of Kv3.4 overexpression on EMT-related marker expressions in mRNA (**F**) and protein (**G**,**H**) levels. (**I–L**) Diff-Quik staining images and the quantified graphs of Control and Kv3.4 overexpressed cells in migration (**I**,**J**) for 24 h and invasion (**K**, **L**) for 48 h (×200). Scale bar 100 μm. The experiments were repeated. ((**J**,**L**): n = 3, (**A**): n = 4, (**C**,**E**,**F**,**H**): n = 5, except, vimentin in (**H**): n = 6). The data represent the mean ± standard error. *p < 0.05; **p < 0.01; ***p < 0.001 (unpaired *t* test).

To explore the role of Kv3.4 expression, we overexpressed Kv3.4 using the *KCNC4* overexpression vector and confirmed it through mRNA and protein analysis in A549 cells (Fig. 4A–C). The overexpression of Kv3.4 apparently exhibited morphological changes. Compared with control, Kv3.4 overexpressed cells had prominently elongated shapes and decreased the circularity index, which is closely related to mesenchymal phenotypes (Fig. 4D,E). Furthermore, we assessed the expression levels of EMT markers in the mRNA and protein levels (Fig. 4F–H). N-cadherin significantly increased both mRNA and protein levels. Vimentin also increased mRNA level but did not alter the protein level. However, the expression of E-cadherin remained continuous in mRNA and protein levels. To examine the functional effects of Kv3.4 overexpression, we performed transwell migration (Fig. 4I,J) and invasion assay (Fig. 4K,L). The numbers of migrated and invaded cells were significantly increased by the overexpression of Kv3.4, compared with control. From these data, we conclude that the overexpression of Kv3.4 acquires mesenchymal characteristics retaining the E-cadherin, which enhances cell migration and invasion.

### The treatment of TGF-β in Kv3.4 overexpressed cells has a synergistic effect on cell migration

**Figure 5:**
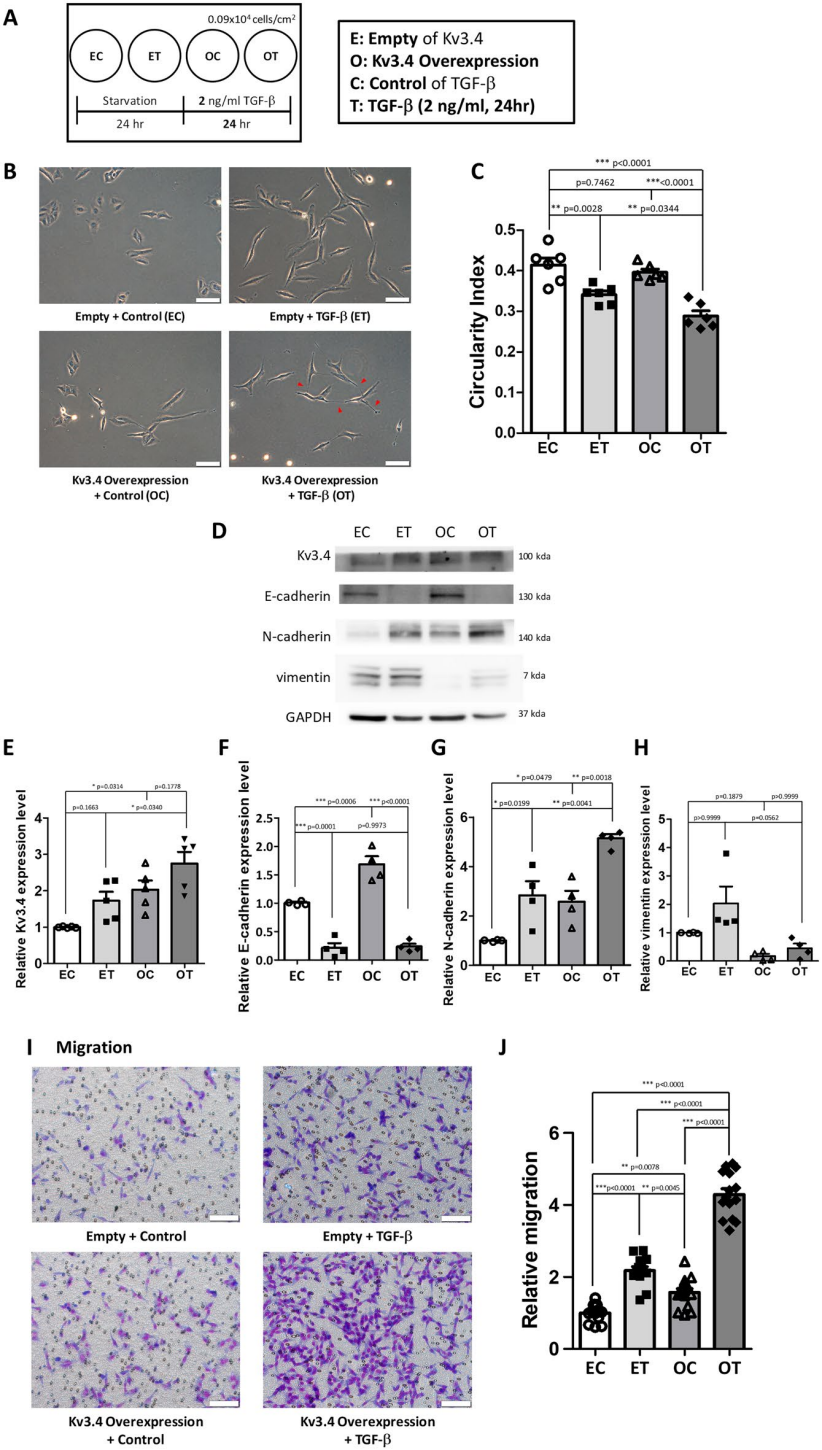
The treatment of TGF-β in Kv3.4 overexpressed cells had a synergistic effect on cell migration. (**A**) Schematic diagram demonstrates the experimental design of TGF-β treatment in Kv3.4 overexpressed A549 cells (left) and the explanations of abbreviations (right). Different from 5 ng/ml of TGF-β treatment for 48 h (Figs. 1, 2), we treated 2 ng/ml of TGF-β for 24 h to confirm the synergistic effect with Kv3.4 overexpression. (**B**,**C**) Representative phase-contrast images (**B**) and the circularity index graph (**C**) show the altered morphology by TGF-β treatment in Empty and Kv3.4 overexpressed cells (×200). Scale bar 100 μm. Arrow pointing to the more elongated form of Kv3.4 overexpressed cells with the TGF-β treatment group. (**D–H**) Western blot data and the quantified graphs show the effects of 2 ng/ml of TGF-β treatment on Kv3.4 overexpressed cells in the protein expression levels of Kv3.4 (**E**), E-cadherin (**F**), N-cadherin (**G**), and vimentin (**H**). (**I**,**J**) Diff-Quik staining images (**I**) and the quantified graph (**J**) of Control and Kv3.4 overexpressed cells with TGF-β treatment in cell migration (×200). Scale bar 100 μm. Experiments were repeated ((**J)**: n = 3, (**F–H**): n = 4, (**E**): n = 5, (**C**): n = 6). The data represent the mean ± standard error. *p < 0.05; **p < 0.01; ***p < 0.001 (one-way ANOVA with Tukey’s multiple comparison test for post-hoc test (**C**,**E–G**,**J**), Kruskal–Wallis test with Dunn’s multiple comparison test for post-hoc test (**H**)). *EC* Empty + Control, *ET* Empty + TGF-β, *OC* Kv3.4 Overexpression + Control, *OT* Kv3.4 Overexpression + TGF-β.

To examine whether TGF-β treatment had a synergistic effect with Kv3.4 overexpression, we treated 2 ng/ml of TGF-β for 24 h after overexpression of Kv3.4 in A549 cells (Fig. 5A). As mentioned before (Fig. 1B), TGF-β treated cells had elongated spindle shapes compared with the cuboidal shapes of control. Kv3.4 overexpressed cells with TGF-β treatment (Fig. 5B,C, **OT**) had also apparently more elongated and spindle-like morphology and reduced the circularity index compared with other control groups: empty control group (Fig. 5B,C, **EC**), only Kv3.4 overexpressed cell group (Fig. 5B,C, **OC**), and empty and TGF-β treatment group (Fig. 5B,C, **ET**). In Figs. 1E and 2B, TGF-β treatment enhanced the expressions of Kv3.4, N-cadherin, and vimentin, while the expression level of E-cadherin was reduced. These results were also confirmed by the treatment of TGF-β in Kv3.4 overexpressed cells, especially in N-cadherin (Fig. 5D–H). To assess the synergistic effect of Kv3.4 and TGF-β in cell migration, we performed the transwell migration assay (Fig. 5I,J). Consistent with these results, Kv3.4 overexpressed cells with TGF-β treatment apparently enhanced the cell migration. Therefore, our results indicate that Kv3.4 could have a considerable synergistic effect with TGF-β.

### Upregulated *KCNC4* expression is associated with poor overall survival in human lung adenocarcinoma and squamous cell carcinoma patients

**Figure 6:**
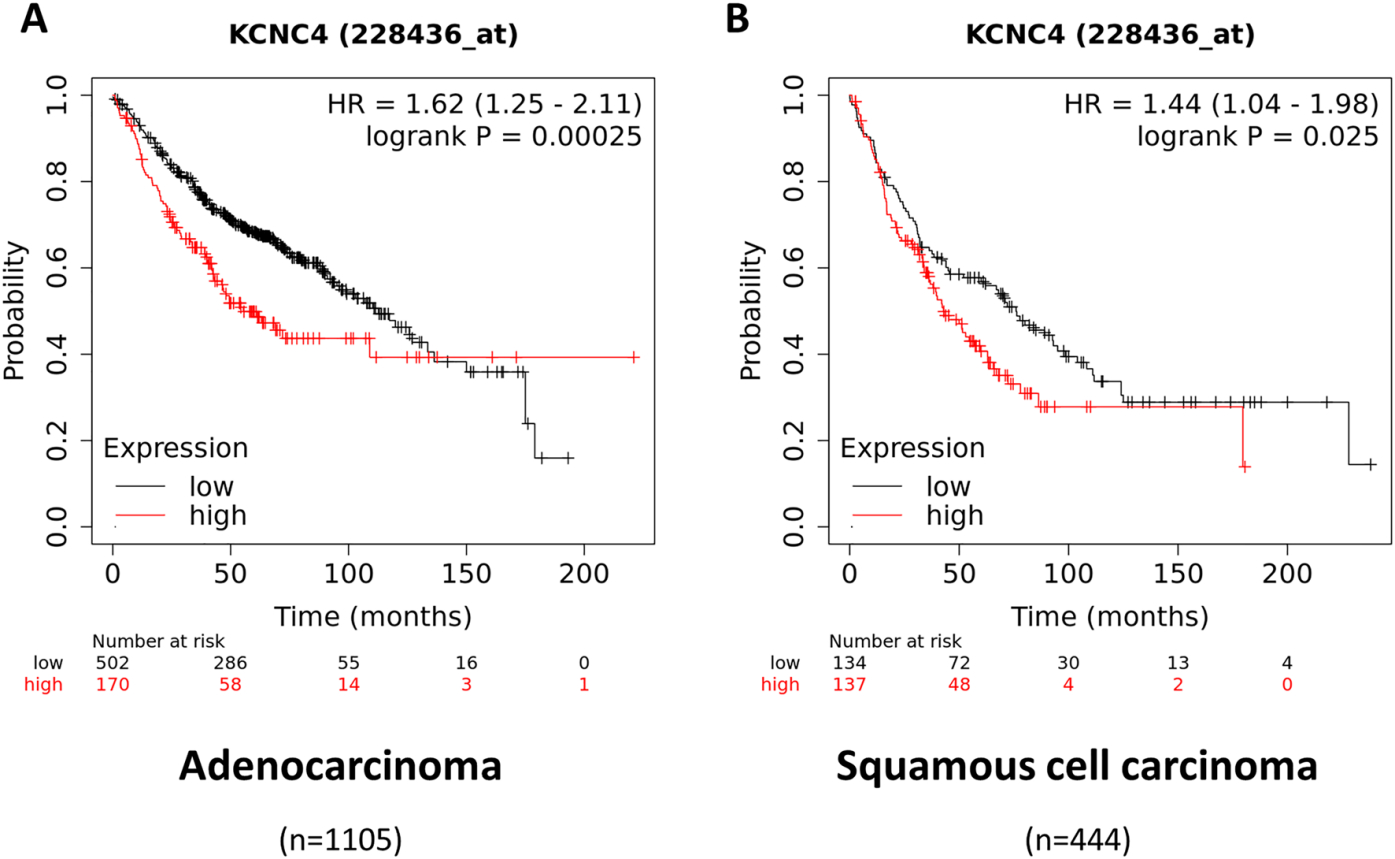
Upregulated *KCNC4* expression is associated with poor overall survival in human lung adenocarcinoma and squamous cell carcinoma patients. KM analysis of the correlation between *KCNC4* expression and overall survival for human lung adenocarcinoma (**A**, n = 1105) and squamous cell carcinoma patients (**B**, n = 444) using the online KM plotter lung cancer database (univariate Cox regression).

To investigate the role of Kv3.4 in human lung cancer, we performed a Kaplan–Meier (KM) analysis of Kv3.4 mRNA levels in lung cancer patients’ overall survival in adenocarcinoma and squamous cell carcinoma (Fig. 6)^25,26^. Lung adenocarcinoma was the most common subtypes in worldwide in 2020, and squamous cell carcinoma accounted for 25% of cases in males and 12% in females, followed by adenocarcinoma (39% for males and 57% for females)^27^. According to the KM plotter of *KCNC4*, high expression levels of *KCNC4* decreased overall survival in both adenocarcinoma (Fig. 6A, hazard ratio (HR) = 1.62, 95% confidence intervals (CI) = 1.25–2.11, log rank P = 0.00025, n = 1105) and squamous cell carcinoma (Fig. 6B, HR = 1.44, 95% CI = 1.04–1.98, log rank P = 0.025, n = 444). Therefore, we conclude that Kv3.4 expression is correlated with survival rates in human lung adenocarcinoma and squamous cell carcinoma.

## Discussion

Kv3.4 is known to be related to cancer metastasis through cell migration and invasion^16^. EMT is a major mechanism for explaining cancer metastasis^4^. However, the relationship between Kv3.4 and EMT remains unclear. In this study, using TGF-β-induced EMT model in A549 cells, we confirmed the effects of cell migration and invasion by the regulation of Kv3.4. Our results showed that TGF-β treatment increased the expression levels of Kv3.4. The knockdown of Kv3.4 blocked the progression of EMT and apparently reduced cell migration and invasion. In contrast, the Kv3.4 overexpressed cells acquired mesenchymal characteristics retaining E-cadherin and promoted cell migration and invasion. Moreover, Kv3.4 overexpression had a synergistic effect with TGF-β treatment. In addition, analyzed by the KM plotter, high expression levels of Kv3.4 were considerably associated with poor overall survival of human lung adenocarcinoma and squamous cell carcinoma patients. Therefore, we conclude that Kv3.4 closely regulates cancer migration and invasion via TGF-β-induced EMT.

EMT is a reversible biological program that converts epithelial cells to mesenchymal cells. In the process of EMT, immotile and apical–basal polarized cells get motile and front–back polarity, which could help cell migration, invasion, and cancer metastasis^1,6,7^. TGF-β is known to be one of the major inducers of EMT^24^. Our data show that TGF-β increased the expression levels of Kv3.4 and had a synergistic effect in Kv3.4 overexpressed cells. It has been known that TGF-β could modulates the expressions and functions of Kv channels. TGF-β increased the expression level of Kv2.1, which induced the maturation of rat primary cerebellar granule neurons^28^. Kv4.2 expression level was increased to transform from rat vascular fibroblasts to myofibroblasts^29^, but decreased in adult rat ventricular myocytes to protect myocytes from hypoxia or ischemia–reperfusion injuries^30^.

We found that the regulation of Kv3.4 induced partial EMT, which supports that Kv3.4 is closely related to cell migration and invasion. EMT is not a process of binary switches from fully epithelial to fully mesenchymal extremes. Instead, cancer cells transit intermediate epithelial/mesenchymal (E/M) states, or partial EMT, during metastatic cascades^6,8,31^. During partial EMT, representative epithelial and mesenchymal markers are exchanged and accompanied by morphological changes, migration and invasion, regulation of various signaling pathways and transcription factors. Therefore, these intermediate states provide advantages to cancer cells associated with phenotypic plasticity and heterogeneity^8^. Previous studies have shown that several Kv channels are involved in EMT, including Kv7.1^20^, Kv10.1^21^ and Kv11.1^14,22,23^. The loss of Kv7.1 promoted EMT related with β-catenin^20^. In contrast, TGF-β-induced EMT enhanced the expression level of Kv10.1 in A549 cells^21^. In addition, the activation of Kv11.1 inhibited EMT and blocked the cancer metastasis by β-catenin in breast cancer^14^. It is also demonstrated that the knockdown of Kv11.1 reversed EMT in colorectal cancer^22^ and Kv11.1 promoted EMT, resulting in the migration and invasion through PI3K/AKT pathway in esophageal squamous cell carcinoma^23^. In the present study, our data also suggest the possibility that Kv3.4 could be related to N-cadherin, a representative EMT marker that is closely involved in cancer metastasis, especially in cell migration^32^. According to previous studies, Kv channels are associated with N-cadherin. Kv1.5 and N-cadherin are colocalized in human cardiac myocytes, which enhances Kv1.5 activity^33^. In addition, the expression of Kv1.5 was apparently reduced in N-cadherin conditional knockout mouse ventricular myocytes^34^. Thus, further studies might be needed to reveal the precise mechanism between Kv channels and EMT, especially Kv3.4.

Recent studies have revealed the diverse roles of Kv3.4. First, Kv3.4 has been linked to the several signaling pathways. As an oxidation- and hypoxia-sensitive channel^35^, Kv3.4 is related to hypoxia-inducing factor 1α (HIF-1α), which binds to the Kv3.4 promoter^36^. Kv3.4 is also associated with calcium signaling, inducing calmodulin/calmodulin-dependent kinase-II axis^37^. Moreover, Kv3.4 is involved to AKT^15^ and extracellular signal-regulated kinase (ERK) signaling pathway^16^ as well. TGF-β is well-known as related to Mothers against decapentaplegic homolog (SMAD) signaling pathway^24,38^. However, our data revealed that the knockdown of Kv3.4 did not affect SMAD signaling (Supplement Fig. 2). Therefore, Kv3.4 might be related to SMAD-independent pathway in TGF-β-induced EMT^38,39^. Next, this study supports that Kv3.4 expression is closely involved with cancer malignancy. In addition to the cancer, Kv3.4 is also associated with various diseases such as cataracts^40^ and Alzheimer’s disease^41,42^. Therefore, using Kv3.4-modulated in vivo model, we expect to be able to reveal the exact mechanisms and possibilities to application to various disease models.

Taken together, we concluded that Kv3.4 is related to TGF-β-induced EMT and cancer migration and invasion in A549 cells. In particular, our results demonstrated that Kv3.4 has a potential to induce partial EMT; First, the regulation of Kv3.4 evokes cellular morphological changes; the Kv3.4 overexpressed cells had prominently elongated shapes. Second, the regulation of Kv3.4 influences the expression levels of EMT-related markers; the knockdown of Kv3.4 reduced the expression levels of mesenchymal markers of N-cadherin, vimentin, and transcription factors (Snail, Slug, Twist), and the overexpression of Kv3.4 increased the mesenchymal traits of N-cadherin and vimentin. Third, the regulation of Kv3.4 had effects on cell migration and invasion; the knockdown of Kv3.4 decreased cell migration and invasion, but the overexpression of Kv3.4 improved cell migration and invasion. Our study demonstrates the role of Kv3.4 is closely involved in cancer malignancy, supported by KM plotter of human lung adenocarcinoma and squamous cell carcinoma data, and acts as an effector with TGF-β. Therefore, the association between Kv3.4 and EMT may provide novel insights into the mechanisms of cancer metastasis, and Kv3.4 could be an efficient prognostic biomarker for cancer malignancy.

## Methods

### Cell cultures

A549 cells were purchased from Korea Cell Line Bank. Cells were maintained at 37 °C with 5% CO_2_ using Roswell Park Memorial Institute (RPMI) 1640 medium (Welgene, Daegu, Korea, #LM011-03) supplemented with 10% fetal bovine serum (FBS, Welgene, #S001-07), and 1% antibiotic–antimycotic solution (Welgene, #LS203-01). When the cells grew sufficiently in a T75 flask (SPL Life Sciences, Gyeonggi-do, Korea, #70075), or 60 mm dishes (SPL, #20060), they were divided into the various culture dishes or plates (SPL).

### Treatment of Kv3.4 siRNA and transient Kv3.4 overexpression

For siRNA experiment, we treated with the 50 nM of *KCNC4* siRNA (Dharmacon, Inc., Colorado, USA, #L-006223-00) for 72 h using Lipofectamine™ RNAiMAX Transfection Reagent (Invitrogen, Massachusetts, USA, #13778075) according to the manufacturer’s instructions. We used Non-targeting control Dharmacon™ siRNA (Dharmacon, Inc., #D-001810-10-50) for control treatment.

For transient overexpression, we transformed the *KCNC4* (NM_004978) vector (Origene, Maryland, USA, #RC212078) to DH5α (RBC Bioscience, New Taipei City, Taiwan, #RH617) cells incubated in LB Agar plates (Difco™ LB AGAR, MILLER (Luria-Bertani), BD Biosciences, New Jersey, USA, #244520) with 25 μg/ml Kanamycin sulfate (Sigma-Aldrich, Missouri, USA, #K1377). We extracted the single-stranded DNA using mini extraction kit (AccuPrep^®^ Nano-Plus Plasmid Mini Extraction Kit, BIONEER, Daejeon, Korea, #K-3111) and confirmed by the sequencing service (Basic sequencing service, BIONICS, Seoul, Korea). After plating A549 cells in 60 mm dishes (SPL), we transfected the 2.5 μg single-stranded DNA with PolyJet™ In Vitro DNA Transfection Reagent (SignaGen^®^ Laboratories, Maryland, USA, #SL100688) following the manufacturer’s instructions. After the transfection, the cells were selected with 1 mg/ml G-418 Disulfate (G-418, AG Scientific, California, USA, #G-1033) and maintained. For the control treatment, we used a control vector (pCMV6-Entry Mammalian Expression Vector, Origene, #PS100001) followed by the same protocols. Cell morphology was observed by CKX53 inverted microscope (Olympus, Tokyo, Japan) and images were taken at 200× magnification.

### Cell circularity index for the quantification of the cell morphology

The morphology of cell was quantified by using the circularity index on the Fiji ImageJ software (National Institutes of Health, Maryland, USA). The circularity index formula is $\small Circularity=4\times \pi \times (area)/{(perimeter)}^{2}$ and the circularity index ranges from 0 to 1; A value = 1 indicates for a round shape, and 0 for an elongated morphology.

### TGF-β treatment to induce the EMT

Using A549 cells, we treated TGF-β (Sigma-Aldrich, #T7039). Before the treatment of TGF-β, cells were starved with 0.1% FBS for 24 h^43^. After starvation, we treated 5 ng/ml TGF-β diluted with 2 mg/ml Bovine Serum Albumin (BSA, Sigma-Aldrich, #A9647) for 48 h with 0.1% FBS. In the case of the combination with TGF-β and Kv3.4 overexpression, Kv3.4-overexpressed cells were also starved with 0.1% FBS with G-418 for 24 h. And then, 2 ng/ml of TGF-β was treated in the cells for 24 h^44^. After the treatment, the cells were prepared for RNA or protein extraction.

### Reverse transcription-polymerase chain reaction (RT-PCR)

RNA extraction for A549 cells was performed using TRIzol™ (Invitrogen, #15596026) according to the manufacturer’s instructions. Isolated RNA (0.5–2 μg) was used to synthesize cDNA using SuperScript™ III Reverse Transcriptase (Invitrogen, #18080093) with Random Primers (Invitrogen, #48190011), Moloney Murine Leukemia Virus (M-MLV) Reverse Transcriptase (Invitrogen, #28025013), 10 mM dNTP (Invitrogen, #10297018) and RNaseOUT™ Recombinant Ribonuclease Inhibitor (Invitrogen, #10777019) under the following reaction conditions: initial step at 25 °C for 5 min and 65 °C for 5 min, and then 37 °C for 2 min, 25 °C for 5 min, 37 °C for 60 min. After that, 70 °C for 15 min and stored at 4 °C.

### Real-time PCR

Real-time PCR was performed on a StepOnePlus™ Real-Time PCR System (Applied Biosystems, California, USA) using a Glyceraldehyde 3-phosphate dehydrogenase (GAPDH) as a reference. The real-time PCR reaction was performed with 2 μl cDNA, 1× TB Green^®^ Premix Ex Taq™ II (Tli RNase H Plus), ROX Reference Dye (TaKaRa Bio, Shiga, Japan, #RR820A), and 10 pM primers (Regular Oligo Service, cosmoGENETECH, Seoul, Korea, Table 1^45^) under the following reaction conditions: initial step at 95 °C for 10 s, and then 40 cycles of cycling processes (95 °C for 5 s and 60 °C for 30 s).

**Table 1:** Real-time PCR primer sets.

Gene	Protein	Primer	Sequence (5’–3’)
*KCNC4*	KCNC4	F	AAT ATC CCA GGG TGG TGA CA
		R	GGT CTT CAA AGC TCC AGT GC
*CDH1*	E-cadherin	F	GAA GGT GAC AGA GCC TCT GGA T
		R	GAT CGG TTA CCG TGA TCA AAA TC
*CDH2*	N-cadherin	F	CCT TTC AAA CAC AGC CAC GG
		R	TGT TTG GGT CGG TCT GGA TG
*VIM*	vimentin	F	TCT ACG AGG AGG AGA TGC GG
		R	GGT CAA GAC GTG CCA GAG AC
*GAPDH*	GAPDH	F	CTC TGC TCC TCC TGT TCG AC
		R	ACG ACC AAA TCC GTT GAC TC

### Western blot analysis

Cells were lysed using radioimmunoprecipitation assay (RIPA) buffer (Thermo Scientific, Massachusetts, USA, #89900) with a 1% protease inhibitor cocktail (Sigma-Aldrich, #P8340) and 10% phosphatase inhibitor (PhosSTOP™, Roche, Basel, Switzerland, #4906845001). After incubating the contents for 10 min in ice, centrifuge at 10,000 rpm at 4 °C for 10 min and collect the supernatant only. The total protein concentration was measured using a bicinchoninic acid (BCA) protein assay kit (Thermo Scientific, #23227).

The quantified proteins (20–30 μg) were loaded on an 8% or 10% acrylamide gel for sodium dodecyl sulfate polyacrylamide gel electrophoresis (SDS-PAGE) and then transferred to a nitrocellulose membrane (Cytiva, GE Healthcare Life Sciences, Illinois, USA, #10600001). Next, 1× TBS-Tween 20 containing 5% nonfat milk (BD Sciences, #232100) was employed to block nonspecific antibody binding, and protein-transferred membranes were probed overnight with commercially purchased primary antibodies targeting the proteins (Table 2). Membranes probed with primary antibodies were incubated with horseradish peroxidase-conjugated goat anti-rabbit (GenDEPOT, Texas, USA, #SA002-500) or anti-mouse secondary antibodies (GenDEPOT, #SA001-500) over 2 h and visualized using a WesternBright™ Quantum™ (Advansta, California, USA, #K-12042-D10). An ImageQuant™ LAS 4000 image analyzer (GE Healthcare Life Sciences) was used to visualize immunocomplexes, and Fiji ImageJ software (National Institutes of Health, Maryland, USA) was used to analyze the data.

**Table 2:** Antibody information.

Antigen	Source	Cat. No	Dilution
Kv3.4	Alomone Labs	APC-019	1:500
E-cadherin	Cell Signaling Technology	3195S	1:500
N-cadherin	Invitrogen	33–3900	1:500
vimentin	Cell Signaling Technology	5741T	1:2000
Snail	Cell Signaling Technology	3879S	1:1000
Slug	Santa Cruz Biotechnology	sc-166476	1:250
Twist	Santa Cruz Biotechnology	sc-81417	1:500
GAPDH	Santa Cruz Biotechnology	sc-32233	1:10,000

### Cell migration assay and invasion assay

The properties of cell migration and invasion were performed using a 24-well hanging insert (SPL, #35224) for transwell migration and invasion assay^46^. Before seeding the cells in a hanging well, cells were pretreated with siRNA or overexpression with Kv3.4 or TGF-β for the treatment group, and control siRNA or control vector or 2 mg/ml BSA for the control group. After cell detachment, the appropriate numbers of cells were plated on the upper side of a cell-permeable membrane with FBS-free media, and media containing 10% FBS were placed on the lower chamber of the membrane. Following incubation of 24 h for migration, the migrated cells were stained using Diff-Quik staining (Sysmex Corporation, Hyogo, Japan, #38721) and imaged by microscope (CKX53, Olympus). For invasion assay, 120 μl of 1.0 mg/ml diluted Matrigel (Corning^®^, Arizona, USA, #356234) was coated on the upper insert membrane for 1 h at 37 °C before cell plating. After plating the appropriate numbers of cells for 48 h, invaded cells were stained using Diff-Quik staining, and imaged. Migrated and invaded cells were randomly photographed in five fields per each membrane and quantified with Fiji ImageJ software (National Institutes of Health).

### Kaplan–Meier analysis of overall survival in human lung adenocarcinoma and squamous cell carcinoma patients in silico

Using the online KM plotter lung cancer database (https://kmplot.com/analysis, 2023.04.20), we generated overall survival KM plotters with *KCNC4* (Affymetrix ID: 228436_at) split patients by ‘auto select best cutoff’ in both lung adenocarcinoma (n = 1105) and squamous cell carcinoma (n = 444) analyzed by univariate cox regression^26^.

### Statistics and reproducibility

Results were representative of at least three independent experiments, and all data were presented as the mean ± standard error. In case of passing the Kolmogorov–Smirnov (KS) normality test, we applied the unpaired *t* test for the statistical analysis of data; if not, we used the Mann–Whitney U test for statistical comparisons between two groups (GraphPad Prism Version 5.0, GraphPad Software, California, USA). Among the groups, we used one-way ANOVA with Tukey’s multiple comparison test for post-hoc test for passing KS normality test; if not, we used the Kruskal–Wallis test with Dunn’s multiple comparison test for post-hoc test (GraphPad Prism Version 6.0, GraphPad Software).

### Supplementary Information

Supplementary Information 1.

Supplementary Information 2.

Supplementary Information 3.

## Data Availability

All data generated in this study are provided in this published article and Supplementary Data. The uncropped original western blot images of the main figures are provided in the file “Supplementary Information”. All other data are available from the corresponding author upon reasonable request.
