# Supplementary material for: Kv3.4 regulates cell migration and invasion through TGF-β-induced epithelial–mesenchymal transition in A549 cells
Source: Sci Rep. 2024 Jan 28;14:2309. doi: 10.1038/s41598-024-52739-4 (PMC10821870; doi:10.1038/s41598-024-52739-4)
Supplement: Supplementary file 3 — Supplementary Information 3. [file 41598_2024_52739_MOESM3_ESM.docx]

**Kv3.4 regulates cell migration and invasion through TGF-β-induced epithelial-mesenchymal transition in A549 cells**

**Supplementary Methods**

**Cell viability test: Cell Counting Kit-8 assay**

The viability of A549 cells with TGF-β or siKv3.4 treatment was determined using a Cell Counting Kit-8 (CCK-8, Dojindo, Rockville, MD, USA, #CK40) according to the manufacturer’s instructions. After the treatment of TGF-β or siKv3.4, CCK-8 was added for 1 hr, and the absorbance at 450 nm was detected using BioTek Synergy HTX Multimode Reader (Agilent Technologies, California, USA). The results were analyzed by GraphPad Prism (Version 5.0, GraphPad Software, California, USA).
